# The modulatory effect of adaptive task-switching training on resting-state neural network dynamics in younger and older adults

**DOI:** 10.1038/s41598-022-13708-x

**Published:** 2022-06-09

**Authors:** Boglárka Nagy, Andrea B. Protzner, Gwen van der Wijk, Hongye Wang, Filomeno Cortese, István Czigler, Zsófia Anna Gaál

**Affiliations:** 1 Institute of Cognitive Neuroscience and Psychology, Research Centre for Natural Sciences, P.O. Box 286, Budapest, 1519 Hungary; 2 Department of Cognitive Science, Faculty of Natural Sciences, Budapest University of Technology and Economics, Budapest, Hungary; 3 Department of Psychology, University of Calgary, Calgary, AB Canada; 4 Hotchkiss Brain Institute, University of Calgary, Calgary, AB Canada; 5 Mathison Centre, University of Calgary, Calgary, AB Canada; 6 Seaman Family MR Centre, Foothills Medical Centre, University of Calgary, Calgary, AB Canada; 7 Institute of Psychology, Eötvös Loránd University, Budapest, Hungary

**Keywords:** Cognitive ageing, Cognitive neuroscience

## Abstract

With increasing life expectancy and active aging, it becomes crucial to investigate methods which could compensate for generally detected cognitive aging processes. A promising candidate is adaptive cognitive training, during which task difficulty is adjusted to the participants’ performance level to enhance the training and potential transfer effects. Measuring intrinsic brain activity is suitable for detecting possible distributed training-effects since resting-state dynamics are linked to the brain’s functional flexibility and the effectiveness of different cognitive processes. Therefore, we investigated if adaptive task-switching training could modulate resting-state neural dynamics in younger (18–25 years) and older (60–75 years) adults (79 people altogether). We examined spectral power density on resting-state EEG data for measuring oscillatory activity, and multiscale entropy for detecting intrinsic neural complexity. Decreased coarse timescale entropy and lower frequency band power as well as increased fine timescale entropy and higher frequency band power revealed a shift from more global to local information processing with aging before training. However, cognitive training modulated these age-group differences, as coarse timescale entropy and lower frequency band power increased from pre- to post-training in the old-training group. Overall, our results suggest that cognitive training can modulate neural dynamics even when measured outside of the trained task.

## Introduction

Cognitive training, i.e., practicing a specific and demanding cognitive task for a few weeks or months, has received attention as a potential method for compensating for or delaying age-related cognitive decline (e.g., processing speed, executive functions, attention, and long-term memory;^1–5^). Although results are mixed in connection with general efficacy (supporting reviews about the positive effects of cognitive training on performance and cognitive functioning in healthy and clinical aging groups^6–9^; opposing reviews about the lack of specific cognitive training-related benefits on cognitive performance and functioning^10–12^), cognitive training has been shown to improve cerebral blood flow, and reduce age associated structural shrinkage, decreases in white matter integrity and functional dedifferentiation^13–17^.

A particular type of cognitive training is adaptive cognitive training, where the trained task’s difficulty level is adjusted based on performance^18^ so that task demand remains slightly higher than the current cognitive capacity. This process is believed to build behavioural and brain flexibility^19^ and lead to larger training effects^20,21^ when compared to cognitive training tasks where there is no variation in the difficulty level^22^. These training-related changes occur even in older participants due to sustained cognitive and brain plasticity like neurogenesis, network reorganization or modified behavioural responses to internal demand or external stimuli^16,17,23–26^.

The cognitive training literature suggests that training has a significant impact mainly within task-related structural, functional and cognitive domains. In our work we examined the influence of adaptive task-switching training^27^ where participants had to make decisions about a letter-number pair (vowel/consonant or odd/even, respectively) based on the colour of the previously presented cue. Participants switched between the letter and number tasks during the training (informatively cued task-switching). Additionally, special characters were shown in some trials in which cases response had to be withhold (no-go stimuli). Behavioural and ERP results showed that after training, older adults performed as well as young adults. Specifically, age-related differences in hits, reaction time and mixing costs (the reaction time difference between mixed and single trials, lower performance in mixed-task repeat trials compared to single-task trials) disappeared for the old-training group, but not the old-control group, while switching costs (the slower reaction time for switch than repeat trials in mixed-task) did not show changes in any of the groups. The P3b component, which was not seen in older adults in pre-training, had comparable amplitudes in old-training and young groups after training, although with different scalp distributions. We also observed training-related enhancement for the N2 amplitude mainly in older adults. Additionally, these behavioural and electrophysiological training-related gains were also seen in different but related tasks. These results suggested that neurocognitive aging processes can be altered, and that these alterations transferred to non-trained tasks.

The main goal of cognitive training interventions is to gain more general neural and cognitive benefits. These more general effects can be detected with the presence of a transfer effect (i.e., training-related changes on non-trained tasks and cognitive functions), as we showed in our adaptive task-switching training. Even more general transfer effects include using knowledge or skills which were learned in one scenario to achieve different goals in another scenario^28^. Meta-analyses and reviews about transfer effects revealed the general presence of near-transfer (training-related improvement in tasks similar to the training task) in cognitive training but results regarding far-transfer (training-related improvement in tasks which are different from the training task in nature or design^29^) are inconsistent (evidence for existing far-transfer effect, e.g.^30–32^; evidence against far-transfer effect, e.g.^33–35^). However, even though the more widespread effects of cognitive training are currently debatable, cognitive training is still an efficient and suitable method for exploring how different cognitive processes and their neural underpinnings can be altered in old age.

The far-transfer effect is not the only measure which can indicate distributed effects of cognitive training. A promising candidate could be the observation of brain changes in resting-state dynamics during cognitive training. Resting state networks show complex yet spatiotemporally structured dynamics that are linked the brain’s functional flexibility through the exploration of possible functional network configurations^36–40^. These dynamics have been related to the effectiveness of different cognitive processes (e.g. attention^41,42^; memory^43^; working memory^44^; cognitive flexibility^45^; cognitive control^46–49^), task-related behavior (e.g.^50–53^), changes in psychiatric disorders^54,55^ and aging (e.g.^56–63^).

One way to examine resting-state neural dynamics is by measuring brain signal variability. Even though signal variability has been defined as neural noise, recent research has begun to emphasize the importance of these temporal fluctuations of brain activity mainly in information processing capacity and functional integrity. Such variability is a crucial characteristic for complex neural interactions, effective and optimal brain functioning, and flexibly responding to internal and external stimuli^37,38,64–67^. At the cellular level, neuronal populations with more diverse firing patterns to the same stimulus (local noise) can encode and generate representations more effectively and adaptively (probabilistic population code;^68–70^) and they can detect weaker signals as well^71–74^. At a more global level, large-scale networks with more dynamic functional connectivity and temporally fluctuating activation patterns show larger information processing capacity and integration, therefore they can maintain optimal learning, environmental adaptation, and flexibility in neural processing^67,75^. In general, a more complex neural system in resting state can model the external environment in a more detailed and effective way, thus is more adaptable^76^.

These dynamic and flexible neural reconfigurations are the main processes behind neural signal complexity. Complexity is linked to the information content of neural activity and its unpredictability which can be measured with entropy. Higher neural complexity is associated with more stochastic and flexible processes within a network while lower neural complexity shows more rigid, regular, and deterministic processes in brain signal dynamics^65,77–82^. Additionally, high neural complexity is accompanied by both high functional integration and segregation, and highly integrated (mutual) information^83,84^. To detect meaningful recurring patterns across a range of different time scales, we employed a multiscale entropy (MSE) measure developed by Costa and colleagues^85,86^. MSE is most suitable for data which has high temporal resolution like EEG, and is sensitive to both linear and nonlinear patterns at multiple time scales^87^. Thus, MSE analysis is a great addition for more commonly applied spectral power analyses which can detect only linear trends in EEG data through measuring the distribution of signal power over different frequencies. Consequently, comparing the results of these two methods can reveal differing contributions from linear and nonlinear processes^88^. MSE can detect temporal complexity and unpredictability at shorter-range/higher frequency (fine scales) and longer-range/lower-frequency (coarse scales) dynamics. Previous studies found that fine scale MSE is connected to local information processing while coarse scale MSE is connected to distributed information processing^79,82,89^.

In the context of healthy aging, several studies have demonstrated increased fine time scale MSE and decreased coarse time scale MSE in older adults as compared to younger adults, which reveals a shift from greater flexibility in distal to greater flexibility in local information processing^65,79,82,90–92^. However, this shift in brain signal complexity is modulated by spatial and cognitive aging dependent variables. Global information processing is more reduced between hemispheres with aging^79^ and the age-related MSE difference is more pronounced at posterior channels^91^. Additionally, the age-related shift from long-range to local processing has been linked to cognitive resilience and better cognitive health, but these results are mixed. Greater fine scale MSE and a greater shift from long-range to local processing predicted better cognitive functions with higher everyday physical activity in older people^93^. However, higher MSE associated with both local and long-range processing was found to be crucial for achieving better performance in perceptual, attentional, and lexical tasks in older individuals^53,92^. Finally, less local and more global information processing was found in clinical aging groups like dementia, Alzheimer’s and Parkinson’s disease patients^89,94–96^.

Corresponding to the age-related differences in temporal neural dynamics, resting-state spectral power also shows significant differences in frequency band power between younger and older individuals^97^. Even though there are inconsistencies in the current literature, in general, low frequencies (delta, theta, alpha bands) show decreased power^91,98–100^, while higher frequencies (beta) show increased power^92,101^ with healthy aging (i.e., and age-related shift in spectral power density;^102–107^). These results are in line with the age-related brain signal complexity changes, namely the decreased power in lower frequency bands with healthy aging can be linked to decreased coarse time scale entropy while the increased power in higher frequency bands can be linked to increased fine time scale entropy^88^.

Earlier studies have revealed notable training-related structural and functional changes in resting-state neural networks mainly in older age-groups, e.g., increased cerebral blood flow, neural activity, white matter integrity, and increased functional connectivity within- and decreased functional connectivity between resting state networks^13,14,108–111^. However, there is only one previous study which applied entropy measures on cognitive training data from older adults^112^, but it was an fMRI and not an EEG study. This experiment had only healthy older participants, and they were randomly assigned to one of the three experimental groups: multi-domain training, single-domain training, and a control group. Resting-state fMRI was recorded at baseline and 1 year after training and two entropy measures were calculated. One was time-domain entropy, which shows the temporal variability of BOLD signals during spontaneous brain activity. Time-domain entropy decreased with aging, and indexed alterations in brain structure and reduced cerebral blood flow^113–115^. The other measure was functional entropy, which detects the relative orderliness of time series patterns (synchronization) among brain regions, thus it is a similar measure to functional connectivity in resting-state networks. Healthy aging was accompanied by increased functional entropy which indicated more widely distributed spontaneous brain activity, thus less differentiated resting-state brain networks and disrupted functional connectivity^116^. Li and colleagues’ cognitive training study revealed lower functional entropy but higher time-domain entropy in both training groups compared to the control group which shows that cognitive training can influence intrinsic brain activity and neural signal complexity.

In our study we examined how adaptive training can modulate general resting-state neural dynamics and information processing, and more specifically, how cognitive training can affect the age-related changes in intrinsic brain activity. We analysed resting-state EEG data from our previously published adaptive task-switching training task^27^. This method is well suited for identifying training-related resting-state neural plasticity in older adults since cognitive control processes, which are crucial for task-switching performance and rely on prefrontal, frontoparietal and basal ganglia activation and functional connectivity, show the most pronounced age-related decline^117–122^. Altogether, we hypothesized that the adaptive task-switching training could modulate the age-related resting-state brain dynamics in both complexity and oscillatory measures revealing distributed training-related effects outside of the trained task.

## Methods

Detailed descriptions of the participants, the task-switching paradigm and the training process have been published in Gaál and Czigler (2018)^27^.

### Participants

We recruited 39 young (18–25 years) and 40 older (60–75 years) women in this study who were divided into control and training groups. Therefore, we had young-control (20 participants, 21.7 ± 1.6 years), young-training (19 participants, 21.4 ± 1.7 years), older-control (20 participants, 66.1 ± 3.1 years) and older-training (20 participants, 65.3 ± 3.3 years) groups. We measured participants’ IQ by the Hungarian version of the Wechsler Adult Intelligence Scale (WAIS-IV^123,124^) in order to detect potential training-related cognitive changes as well as to rule out those subjects whose IQ score was too low. Our groups’ IQ scores (mean ± SD) and their difference from the average IQ score were the following: IQ (young-control) = 109.4 ± 14.4, t(19) = 2.92, *p* = 0.009; IQ (young-training) = 107.9 ± 12.3, t(18) = 2.80, *p* = 0.012; IQ (old-control) = 120.1 ± 15.8, t(19) = 5.69, *p* < 0.001; and IQ (old-training) = 117.9 ± 16.7, t(19) = 4.79, *p* < 0.001. Additionally, older participants had higher IQ scores as compared to younger ones (main effects ANOVA: F(1, 76) = 9.543, *p* = 0.003). Every participant was right-handed, had normal or corrected-to-normal vision, and had no history of neurological or psychiatric disorder.

The protocol was approved by the United Ethical Review Committee for Research in Psychology (EPKEB, Hungary) and the study and all of the applied methods were conducted in accordance with the Declaration of Helsinki. A written informed consent was obtained from all participants, and they were paid for their contribution.

### Procedure

**Figure 1:**
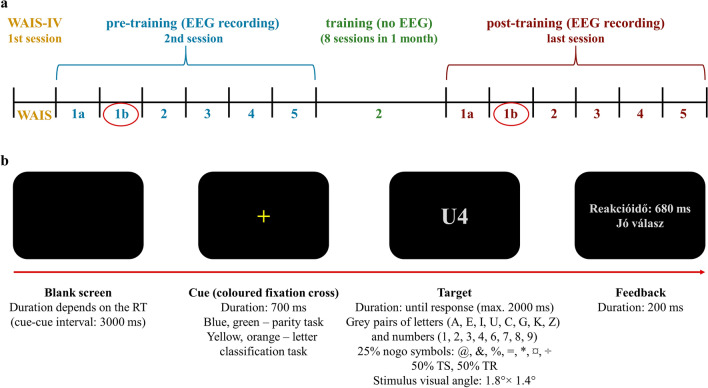
Experimental design of the adaptive task-switching training study^27^. (**a**) The structure of the executed tasks. Sitting with eyes closed (1a) and open (1b) for 2 min each, (2) informatively cued task-switching paradigm with no-go stimuli—letter classification and parity task (trained task), (3) non-informatively cued task-switching paradigm with no-go stimuli—letter classification and parity task, (4) informatively cued task-switching paradigm—colour and shape classification, and (5) Attentional Network Test. In the training sessions the difficulty of the informatively cued task-switching paradigm with no-go stimuli was modified block by block based on performance. The resting-state EEG with eyes open from pre- and post-training sessions (1b) were analysed in this study. (**b**) The schema of the informatively cued task-switching paradigm with no-go stimuli—letter classification and parity task (trained task). TR: task-repeat, TS: task-switching.

During the first session in the laboratory, each participants’ health status, habits, demographic data, and IQ were documented. Electroencephalogram (EEG) was recorded in the second and final sessions which were conducted one month apart. These EEG data were part of a larger project that included eyes closed and open as well as different task-related recordings. The full protocol was described in detail by Gaál and Czigler (2018)^27^, and a detailed description of the experimental and training procedure can be seen in Fig. 1. Particiapnts performed several different tasks during EEG recording, in the following order: (1) eyes closed and open resting-state for 2 min each, (2) informatively cued task-switching paradigm with no-go stimuli—letter classification and parity task (this is the trained task), (3) non-informatively cued task-switching paradigm with nogo stimuli—letter classification and parity task (this task was used for for detecting near-transfer effects in the original study, and it was not trained), (4) informatively cued task-switching paradigm—colour and shape classification (this task was used for detecting near-transfer effects in the original study, and it was not trained), and (5) Attentional Network Test (this task was used for detecting far-transfer effects in the original study, and it was not trained). Each task began with a practice block where participants were given the instructions both orally and in writing.

In connection with the current study’s goals, we are focusing on the trained, informatively cued task-switching paradigm with no-go stimuli, thus we describe it in more detail here (Fig. 1). Every trial started with a coloured cue where yellow and orange cues were assigned to the letter classification task (vowel/consonant decision) and blue and green cues to the number classification task (odd/even decision). After 700 ms, a letter-number target pair was shown in the centre of the screen. Participants pressed the button of a game pad with their left or right index finger based on the mapping of the letter and parity task which was counterbalanced. In all trials except four per block, the two stimuli required opposite button presses. In 25% of the trials, special characters were shown as targets where response had to be withheld (nogo trial). The target was shown until response (maximum 2,000 ms) and feedback was displayed post-response on the screen for 200 ms. The paradigm had the following restrictions: (1) cue colour was never repeated in successive trials, (2) target stimuli were presented in pseudorandom order with no repetition on successive trials, (3) 50% switch probability, (4) two nogo trials could not follow each other, (5) maximum three switch or repeat trials could follow each other. Two blocks of single letter, and two blocks of number classification trials were presented at the beginning of each session (104 trials each). These were followed by 10 blocks of mixed trials (50 trials per block).

While the control groups participated only in the aforementioned three sessions (passive control), the training groups completed eight 1-h adaptive training sessions of the informatively cued task-switching paradigm with no-go stimuli. The difficulty level was personalized in each block based on the current performance of each participant and it varied based on the number of tasks (1–4) and the number of cues for each task (1–2): (1) parity task—odd/even number decision (blue and green cue), (2) letter classification task—vowel/consonant decision (yellow and orange cue), (3) magnitude task—less/more than five decision (lilac and pink cue), (4) letter classification task—lower/uppercase decision (red and brown cue). This adaptive cognitive training took place between the second and last session for every participant who underwent training. Thus, we refer to these sessions as pre-training (first and second sessions) and post-training (last session).

For the current analyses, we used the pre-training open-eyed resting-state EEG data for detecting general age-related differences in the neural network dynamics, as well as pre- and post-training open-eyed resting-state EEG data in order to examine training-related changes in the neural network dynamics and its age-related modifications. Additionally, we used the informatively cued task-switching with no-go stimuli (letter/parity decision) task’s cue- and target-locked event-related potentials (ERPs) from pre- and post-training conditions for detecting those electrodes where training-related changes could be detected. We focused on this task because it was used in the adaptive training, thus its cue-and target-locked ERP data could reveal training-related changes in the younger and older age-groups connected to anticipatory and task-execution processes, respectively. More detailed description of these analyses are provided in the “EEG acquisition and preprocessing” and “PLS analysis of task data to identify training-relevant electrodes” sections below.

### EEG acquisition and preprocessing

Electrophysiological recording was performed in an electrically and acoustically shielded room. In the adaptive training sessions, stimuli were presented by Presentation software in the centre of a monitor (LG 915FT Plus 19”; 800 × 600 pixels; 60 Hz refresh rate) at a viewing distance of 125 cm. Continuous EEG was recorded by NuAmps amplifiers (bandpass: DC-70 Hz) using NeuroScan 4.4 software (Compumedics, Victoria, Australia; sampling rate: 1000 Hz). Thirty-five passive Ag/AgCl electrodes were placed on Fp1, AFz, Fp2, F7, F3, Fz, F4, F8, FT9, FC5, FC1, FC2, FC6, FT10, T7, C3, Cz, C4, T8, TP9, CP5, CP1, CP2, CP6, TP10, P7, P3, Pz, P4, P8, PO9, O1, Oz, O2, PO10, referenced to the tip of the nose, with FCz as ground. Vertical and horizontal eye movements were recorded by electrodes placed above and below the left eye (VEOG) and in the outer canthi of the eyes (HEOG). The impedance of the electrodes was kept below 10 kΩ.

In order to preprocess raw resting-state EEG data offline for further analyses, functions of EEGLAB v.14.1.0b^125^ were applied for finite impulse response band-pass filtering at 0.5–40 Hz followed by independent component analysis (ICA) for removing ocular (i.e., eye blinks, horizontal eye movements), muscle and cardiac artefact. Data were segmented into 2500 ms epochs continuously and epochs were rejected if they had a voltage change larger than 100 µV between their minimum and maximum. The average number of rejected epochs were the following (mean ± SD): young-control pre-training: 2.9 ± 4.1, young-control post-training: 5.2 ± 9.7, young-training pre-training: 6.8 ± 10.9, young-training post-training: 7.9 ± 11.0, old-control pre-training: 7.7 ± 12.7, old-control post-training: 4.8 ± 7.8, old-training pre-training: 2.7 ± 4.6, old-training post-training: 2.2 ± 3.6. As for the ERP analysis, the preprocessing of raw task-related EEG data was started with 0.1–30 Hz filtering followed by segmentation (cue-locked ERPs: − 100 ms to 700 ms relative to cue onset; target-locked ERPs: -100 ms to 1000 ms relative to target onset), baseline correction (using prestimulus interval) and automatic artifact rejection (epochs were rejected if they had a voltage change larger than 80 µV between their minimum and maximum).

### Multiscale entropy estimation of brain signal variability

Full details of multiscale entropy (MSE) and its relevance for the analyses of signal complexity are provided in studies by Costa et al.^85,86^. The MSE method calculates sample entropy as a measure of the unpredictability of the EEG signal at different timescales, where greater MSE values represent greater entropy. The calculation of MSE involves two steps. First, data are resampled into different timescales, then the sample entropy for each time series is calculated. For each timescale, data points are averaged within non-overlapping windows of the scale’s length. For example, the original time series corresponds to scale 1 (i.e., 1 ms windows in the context of our 1000 Hz sampling rate), scale 2 is averaged over two time points (i.e., 2 ms windows), and so on. After that, sample entropy of each coarse-grained time series for each epoch is calculated, measuring predictability by evaluating the appearance of repetitive patterns based on two parameters: the pattern length (m) and the tolerance level (r). The pattern length indicates how many consecutive data points are used for pattern matching and the tolerance level determines the maximal absolute amplitude difference between two data points to consider them matching. Therefore, sample entropy reflects the probability that two sequences that match on the first *m* data points will also match on the next data point. We calculated MSE for each epoch and each participant using the algorithm available at www.physionet.org/physiotools/mse/, with parameter values m = 2^126^ and r = 0.5^127^. These values were chosen based on earlier aging studies^79,88,91^. The length of the time series was 2500 data points (corresponding to 2500 ms epochs at 1000 Hz sampling rate). To ensure reliable MSE estimation, we included only those timescales for which we had at least 50 samples. Thus, for each participant, MSE estimates were obtained for each epoch and electrode at each timescale from 1 to 50 (or 1–50 ms windows) where lower values represent fine timescales and higher values represent coarse timescales. Subsequently, MSE values were averaged across all trials in every participant separately to obtain mean MSE for each timescale and electrode in the pre- and post-training conditions.

### Spectral power density estimation

We calculated spectral power density (SPD) in order to compare MSE and SPD results which could allow us to relate MSE measures to the frequency content of the EEG signal and to evaluate if age- and training-related differences are driven by linear (assessed by both MSE and SPD) or nonlinear (assessed only by MSE) dependencies in the data^88^. SPD of the signal was calculated using fast Fourier transform on single trial data. The signal was first normalized (mean: 0, SD: 1) to deal with age-related global signal power differences (e.g.,^100,105,128,129^). Relative contributions of different frequency bands to the total spectral power were calculated based on normalized data. Given a sampling rate of 1000 Hz and 2500 time points (equal with 2500 ms) per trial, the frequency resolution was 0.4 Hz. Single-trial estimates were averaged across trials in every participant to obtain mean SPD for resting-state condition.

### Partial least squares analysis

Partial least squares (PLS) analysis^130–133^ is a data-driven multivariate analysis technique which operates on the entire data structure at once, extracting the patterns of maximal covariance between brain signals and groups/conditions. Here, PLS analyses were applied first on pre-training resting-state MSE and SPD measures for assessing general age-related differences in every electrode. In order to examine training-related changes in neural network dynamics, the first step was to run PLS analyses on cue- and target-locked ERP data of the trained reference task from our previous work (informatively cued task-switching with no-go stimuli—letter/parity decision;^27^). These analyses allowed us to identify those electrodes where significant training-related changes could be detected (more details in the “PLS analysis of task data to identify training-relevant electrodes” section in “Results”). Therefore, a task-related (neural) network was identified, and we selected these specific electrodes for our main analyses on resting state EEG data. Thus, we examined whether adaptive training could engender more general changes in neural network dynamics at those electrode sites where task-specific training effects could be detected. Altogether, we applied PLS analyses to assess training-related changes and its age-related differences in spatiotemporal distributions of resting-state MSE and SPD measures.

PLS operates on the covariance between brain signal (as measured by MSE or SPD) and the experimental design across participants to identify a new set of variables (so-called latent variables or LVs) that optimally relate the two sets of measurements. Each LV contains three vectors: design saliences, electrode saliences, and singular values. Design saliences indicate the degree to which each condition within each group is related to the brain signal pattern identified in the LV. Design saliences should be interpreted as the optimal contrast that codes the effect depicted in the LV. Electrode saliences are numerical weights that identify a particular pattern of electrodes and timescales/frequencies that are most related to the group and condition effects expressed in the LV. The singular values represent the strength of the effect expressed by the LV (i.e., the covariance between the contrast and the MSE or SPD pattern). The PLS analysis is similar to other multivariate techniques, such as principal component analysis (PCA), in that the algorithm extracts LVs explaining the covariance between conditions and brain activity in order of the amount of covariance explained, with the LV accounting for the most covariance extracted first.

Statistical assessment in PLS is performed across two levels. First, the overall significance of each LV is assessed with permutation testing^134^ where the group or condition labels are re-assigned for each subject. An LV was considered significant and different from random noise if the observed singular value exceeded the permuted singular value in more than 95% of the permutations (corresponding to *p* < 0.05). Second, bootstrap resampling^135,136^—drawing randomly with replacement from subjects in every group—is used to estimate confidence intervals around electrode timescale or frequency weights in each LV as well as assessing their relative contribution and the stability of their relation with experimental groups. No corrections for multiple comparisons are necessary because the electrode saliences are calculated in a single mathematical step on the whole brain. In this study 500 permutations and 500 bootstrap samples were tested. For the brain data, the plotted bootstrap ratios (ratio of the individual weights over the estimated standard error) were proportional to z scores, with a minimum threshold of 2.0 corresponding approximately to a 95% confidence interval or *p* < 0.05.

**Figure 2:**
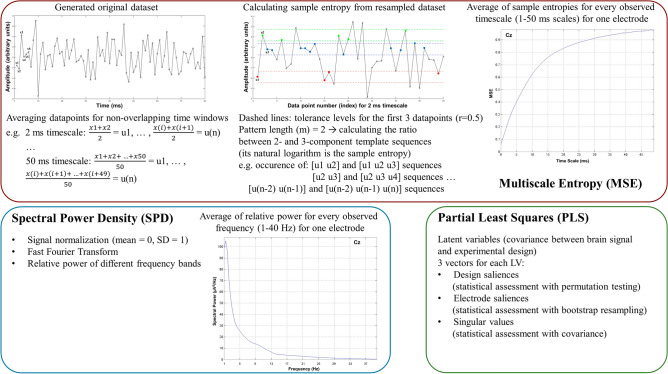
Illustration and summary of the applied analysis methods.

**Figure 3:**
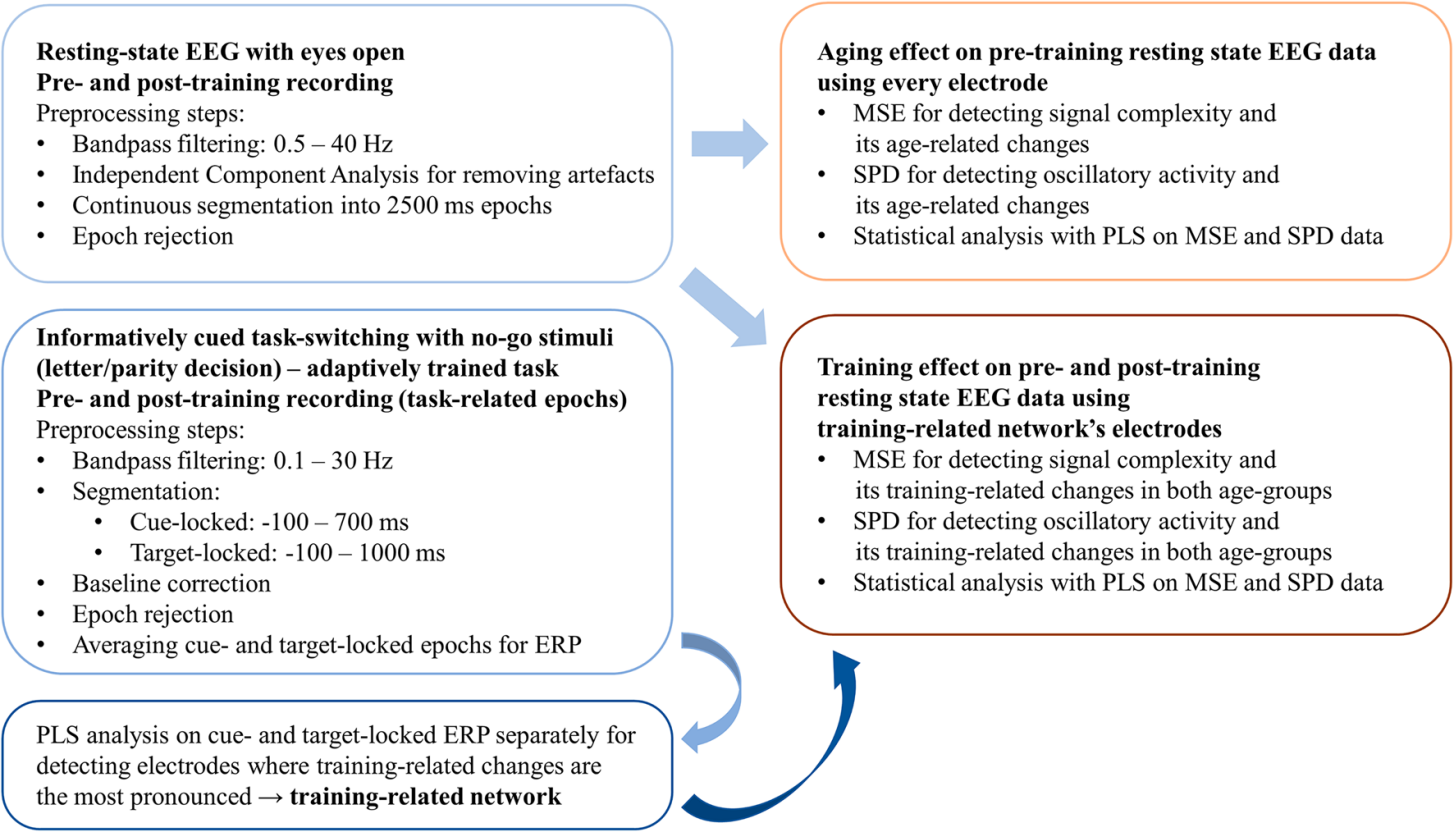
Flowchart and summary of the EEG data processing steps. ERP: event-related potential, MSE: multiscale entropy, SPD: spectral power density, PLS: partial least squares.

For the visualization of our analyses, the Scientific colour map vik^137^ is used in this study to prevent visual distortion of the data and exclusion of readers with colour-vision deficiencies^138^. A summarized illustration of the applied analysis methods can be seen in Fig. 2, and the flowchart of the EEG data processing steps is shown in Fig. 3.

## Results

### General aging effects in pre-training resting-state MSE

**Figure 4:**
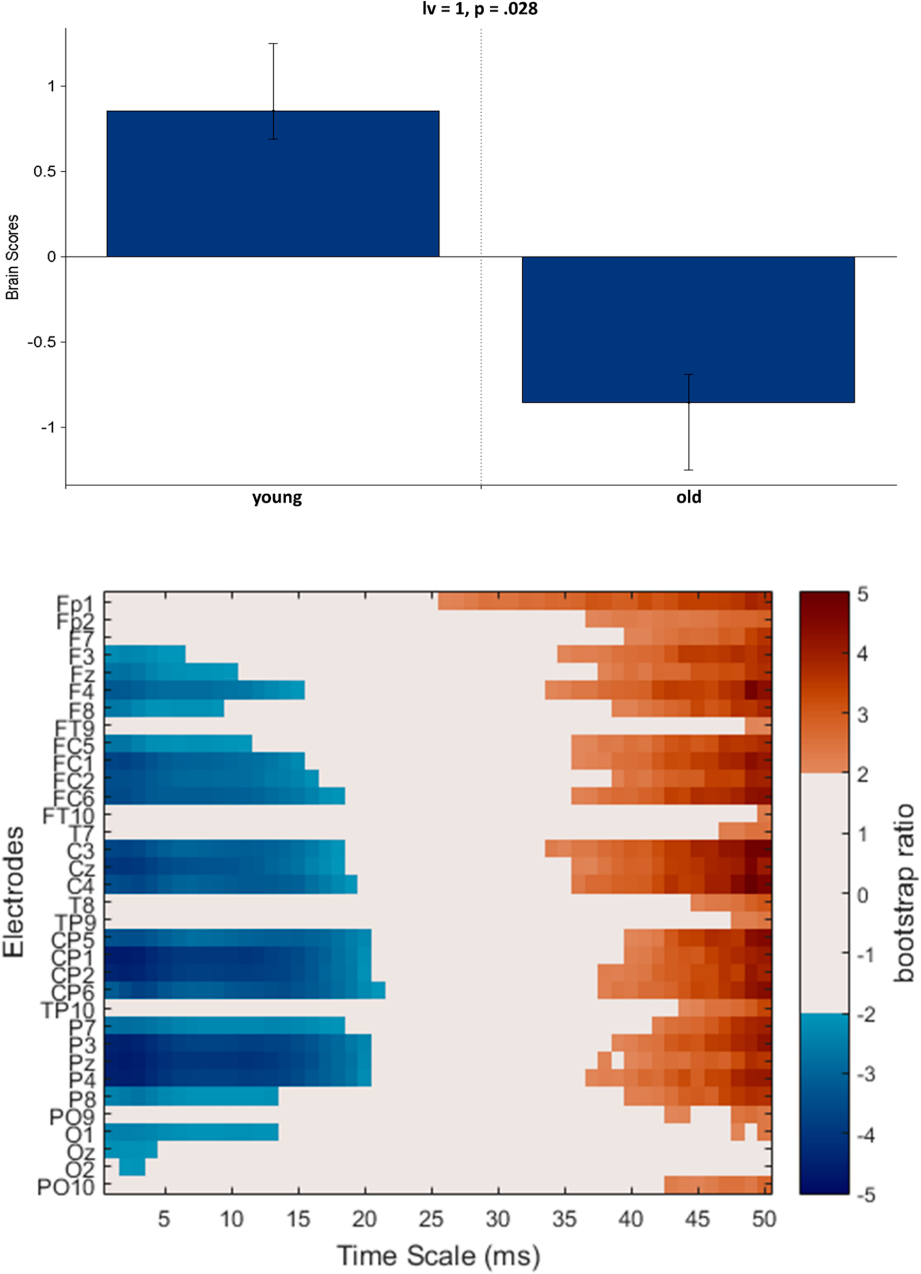
PLS analysis results for the general aging effect on MSE (using the pre-training resting state in our two age-groups). (Top) The bar graph depicts the contrast between age-groups that was significantly expressed across electrodes and timescales as determined by permutation tests, with error bars denoting 95% confidence intervals. (Bottom) The bootstrap ratio map illustrates the electrodes and timescales at which the contrast displayed in the bar graphs was most stable. Values represent the ratio of the individual electrode weights and the bootstrap-derived standard error (roughly z scores, thresholded at 2.0 which corresponds approximately to *p* < 0.05). Positive values are plotted in warm clours and indicate timescales and electrodes showing decreases from young to old age-group in resting state MSE. Negative values are plotted in cool colours and denote increases from young to old age-group in resting state MSE.

Before examining training-related changes, we were interested in the general age-related differences in neural network dynamics prior to training, thus task PLS analysis was performed on pre-training resting-state MSE using our full young and older age-group and all electrode sites. One significant LV was identified which revealed general age-related resting-state MSE differences (*p* = 0.028, Fig. 4). This LV showed decreased coarse temporal scale MSE (scales: 35–50 ms) and increased fine temporal scale MSE (scales: 1–20 ms) from young to old age-groups at nearly all electrode sites (no difference at occipital electrodes and less robust effects at frontal electrodes).

### General aging effects in pre-training resting-state SPD

**Figure 5:**
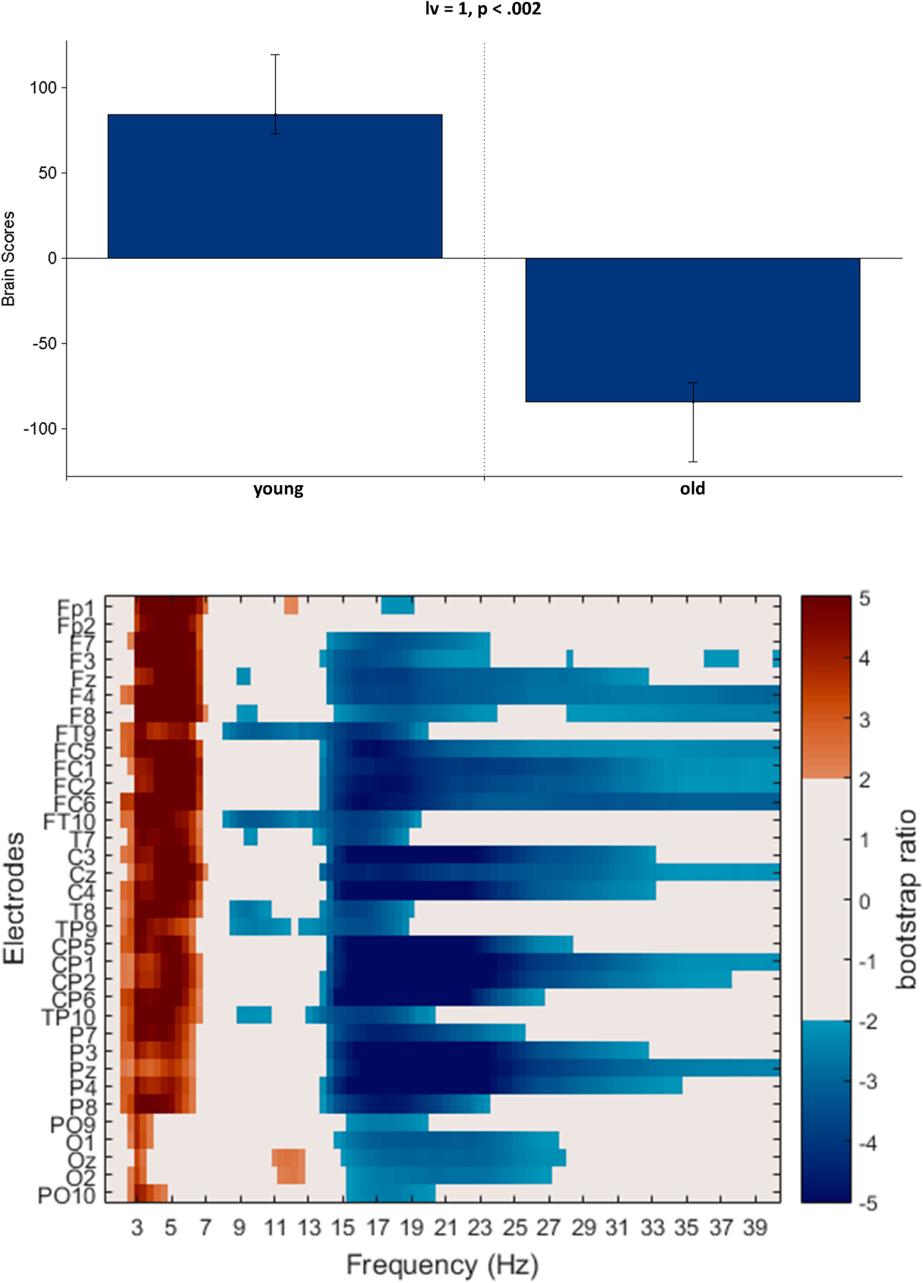
PLS analysis results for the general aging effect on SPD (using the pre-training resting state in our two age-groups). (Top) The bar graph depicts the contrast between age-groups that was significantly expressed across electrodes and frequencies as determined by permutation tests, with erros bars denoting 95% confidence intervals. (Bottom) The bootstrap ratio map) illustrates the electrodes and frequencies at which the contrast displayed in the bar graphs was most stable. Values represent the ratio of the individual electrode weights and the bootstrap-derived standard error (roughly z scores, thresholded at 2.0 which corresponds approximately to *p* < 0.05). Positive values are plotted in warm colours and indicate frequencies and electrodes showing decreases from young to old age-group in resting state SPD. Negative values are plotted in cool colours and denote increases from young to old age-group in resting state SPD.

We analysed general age-related differences in resting-state SPD as well with PLS using pre-training data. One significant LV was identified which revealed age-related resting-state SPD differences (*p* < 0.002, Fig. 5). This showed increased power in beta (15–30 Hz) and to a lesser extent in gamma (≥ 30 Hz) frequency bands and decreased power in delta and theta (1–7 Hz) frequency bands from young to older age-group throughout all of our analysed electrodes (these effects were less robust at occipital electrodes).

### PLS analysis of task data to identify training-relevant electrodes

**Figure 6:**
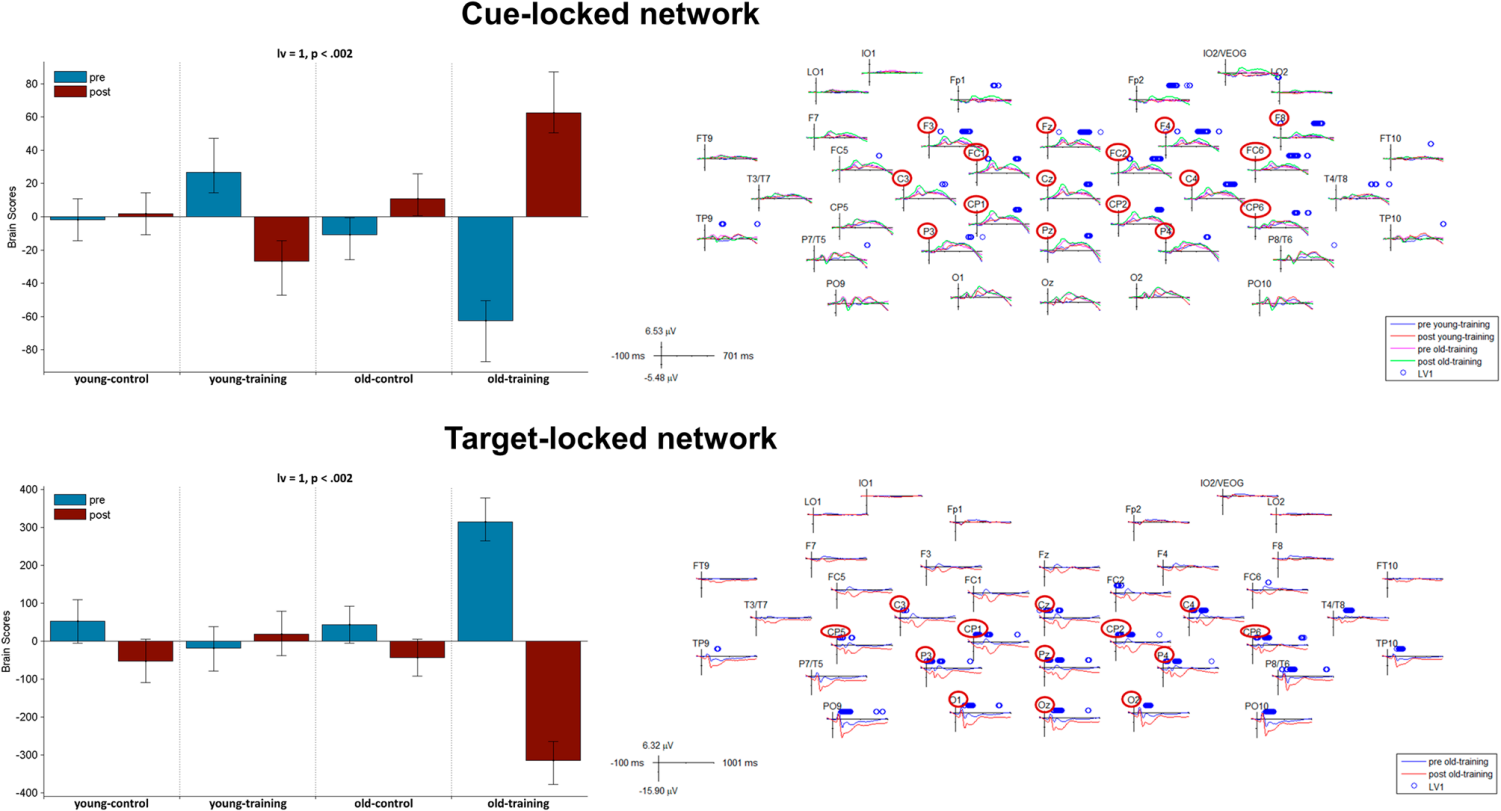
PLS analysis results on cue-locked (top row) and target-locked (bottom row) ERP for the group by condition effect (pre- vs. post-training task). On the left side, the bar graph depicts the contrast between experimental groups across age and training conditions that was significantly expressed across electrodes and timepoints as determined by permutation tests, with error bars denoting 95% confidence intervals. On the right side, the ERPs for all of the electrodes used in the original study are shown and the blue circles represent those timepoints where the detected contrast (LV) reaches high significance as determined by bootstrapping. The cue-locked waveforms show the trained task’s ERPs for the young-training group’s pre-training (blue) and post-training (red) as well as the old-training group’s pre-training (magenta) and post-training (green) conditions and the blue circles represent those timepoints where the bootstrap ratio is larger than 4.5. The target-locked waveforms show the trained task’s ERPs for the old-training group’s pre-training (blue) and post-training (red) conditions, and the blue circles represent those timepoints where the bootstrap ratio is larger than 8.1. Electrodes highlighte with red circles constitute the training-relevant electodes that were used for further analyses with resting-state MSE and SPD.

Using ERP data collected during task performance (i.e., not the resting-state data used in our current analyses), we identified training-relevant regions where significant training-related changes could be detected in cue-locked and target-locked ERPs^27^. Our PLS analysis revealed one significant LV where the training effect could be detected in each of the cue- and target-locked analyses (both *p* < 0.002, Fig. 6). In the cue-locked analysis, the opposite training-effect was identified in the young- and older-training groups (decreased amplitudes in the young-training group and increased amplitudes in the older-training group from pre- to post-training) while for the target-locked ERP, the training-effect (significant changes in target-locked ERP from pre- to post-training) was shown only in the older-training group. Based on electrodes in which stable cue- and target-locked effects were identified, we defined a set of task-related electrodes including: F3, Fz, F4, F8, FC1, FC2, FC6, C3, Cz, C4, CP5, CP1, CP2, CP6, P3, Pz, P4, O1, Oz, O2. Peripherally located electrodes were excluded. Our resting-state analyses (aging and training effects in resting-state MSE and SPD) were executed using these 20 channels.

### Aging and training effects in resting-state MSE

**Figure 7:**
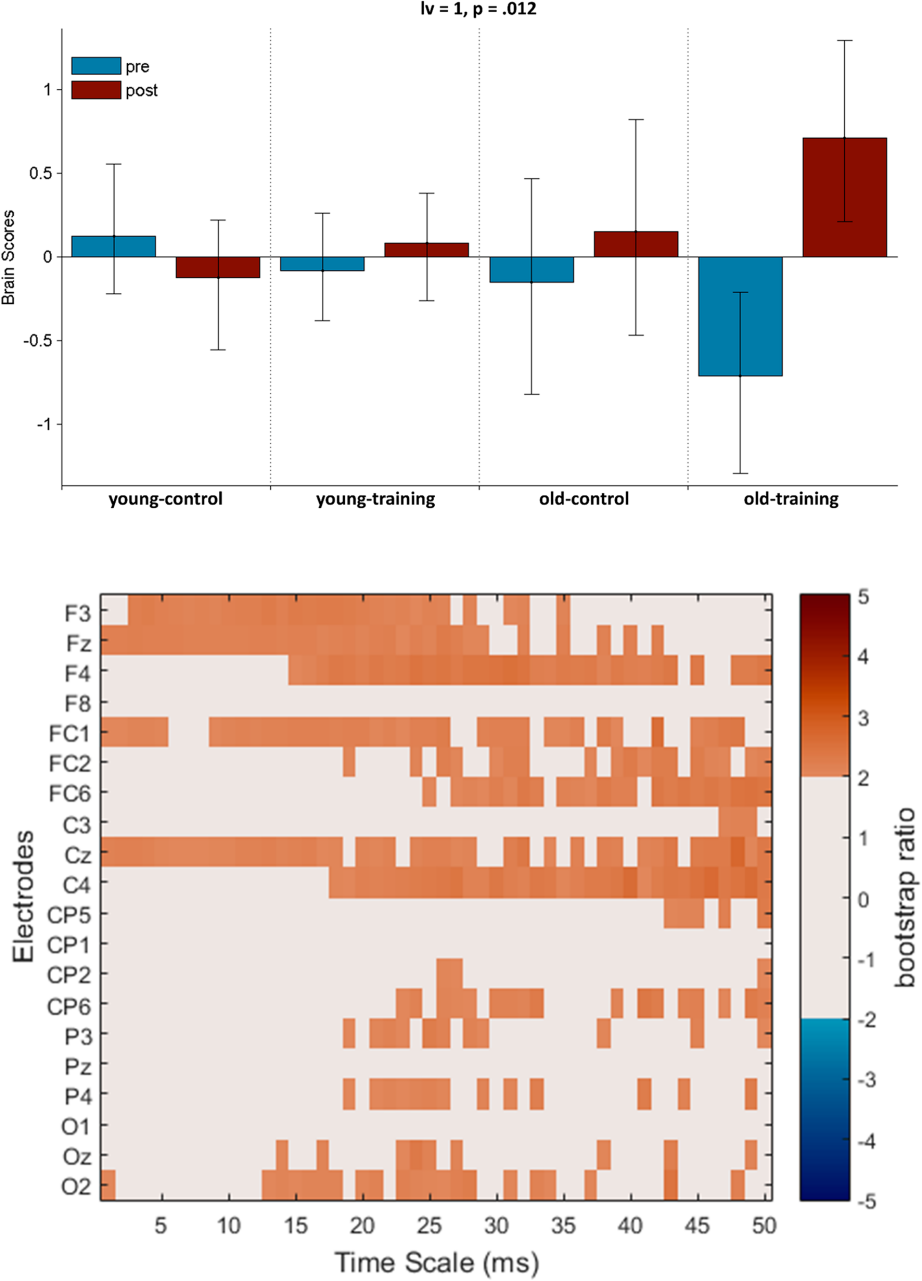
PLS analysis results for the group by condition effect (pre- vs. post-training resting state) on MSE. (Top) The bar graph depicts the contrast between experimental groups across age and training conditions that was significantly expressed across electrodes and timescales as determined by permutation tests, with error bars denoting 95% confidence intervals. (Bottom) The bootstrap ratio map illustrates the electrodes and timescales at which the contrast displayed in the bar graphs was most stable. Values represent the ratio of the individual electrode weights and the bootstrap-derived standard error (roughly z scores, thresholded at 2.0 which corresponds approximately to *p* < 0.05). Positive values are plotted in warm colours and indicate timescales and electrodes showing increases from pre- to post-training in resting state MSE. Negative values are plotted in cool colours and denote decreases from pre- to post-training in resting state MSE.

We examined age-related differences in potential training-related effects with a task PLS analysis performed on pre-training and post-training resting-state MSE. One significant LV was identified which revealed training-related resting-state MSE changes in the older-training group (*p* = 0.012, Fig. 7). This showed increased coarse temporal scale MSE (scales: 20–50 ms) from pre- to post-training mostly at midline and right fronto-central electrode sites in the older-training group. To a lesser extent, increased fine temporal scale MSE (scales: 1–20 ms) also could be detected in the same group post-training at midline and left fronto-central areas. We did not identify any training-related MSE changes in the young-training group or the control groups.

### Aging and training effects in resting-state SPD

**Figure 8:**
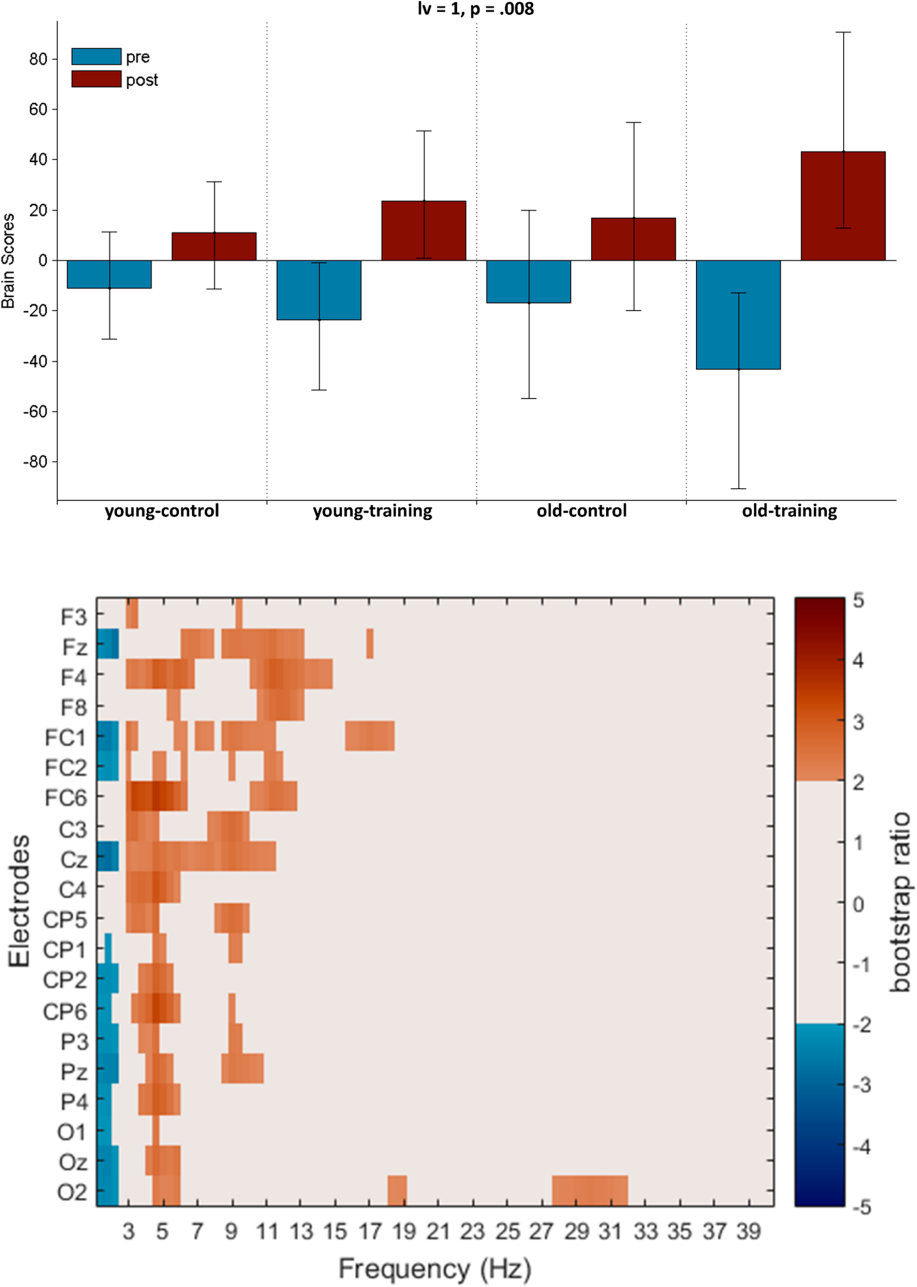
PLS analysis results for the group by condition effect (pre- vs. post-training resting state) on SPD. (Top) The bar graph depicts the contrast between experimental groups across age and training conditions that was significantly expressed across electrodes and frequencies as determined by permutation tests, with error bars denoting 95% confidence intervals. (Bottom) The bootstrap ratio map illustrates the electrodes and frequencies at which the contrast displayed in the bar graphs was most stable. Values represent the ratio of the individual electrode weights and the bootstrap-derived standard error (roughly z scores, thresholded at 2.0 which corresponds approximately to *p* < 0.05). Positive values are plotted in warm colours and indicate frequencies and electrodes showing increases from pre- to post-training in resting state SPD. Negative values are plotted in cool colours and denote decreases from pre- to post-training in resting state SPD.

We analysed age-related differences in potential training-related effects from pre-training to post-training resting-state SPD with a task PLS. We identified one significant LV which revealed training-related resting-state SPD changes in the young- and older-training groups (*p* = 0.008, Fig. 8). There was increased power in high delta and theta frequency bands (3–7 Hz) from pre- to post-training across all task-related electrodes but mainly at midline and right centro-parietal electrodes in the young- and old-training groups. Moreover, increased alpha power (8–14 Hz) was found at fronto-central electrode sites by training in both age-groups. No changes were detected from pre- to post-training in the young- and old-control groups.

## Discussion

The purpose of this study was to investigate the influence of age on adaptive task-switching training-related changes in resting-state neural activity and brain dynamics. We used resting-state EEG data from our previously published training study, and we ran multiscale entropy (MSE) and spectral power density (SPD) analyses in order to measure brain signal complexity and oscillatory activity, respectively. We compared control and training groups in both younger and older adults for detecting age-related differences in the training-effect, and found increased complexity following training mostly in connection with broader and long-range neural processing (increased coarse scale MSE and lower frequency power) for the old-training group.

Firstly, we established how our young and old age-groups’ pre-training brain dynamics differed prior to training, and whether these results are consistent with previous research. This pre-training comparison was important for evaluating training-related changes as well, by allowing us to identify baseline differences between our age-groups to guide our evaluation of the post-training results. We replicated previous findings in connection with intrinsic brain signal complexity and oscillatory activity differences with aging. Increased fine temporal scale MSE (1–20 ms) and decreased coarse temporal scale MSE (35–50 ms) was detected in older compared to younger adults (similar results in e.g.:^65,79,82,90–92^). As in previous work, we interpret this result to reflect a shifting balance between local and long-range processing flexibility, where older adults show an increased reliance on local information processing and decreased long-range interactions with distant brain regions as compared to young adults. We also found increased high frequency power (beta, gamma) and decreased low frequency power (delta, theta) in older compared to younger adults (similar results in e.g.:^102–107^). These age-related resting-state changes occured broadly (closer to midline) but they were most pronounced at central and parietal channels, followed by frontal channels, and the weakest at occipital channels. This spatial pattern is somewhat in line with previous studies, since age-related changes in resting-state slow wave power is most prominent in frontal, midline and parietal regions^100,102^ while changes in beta power occurs in central regions^102,104^. Additionally, studies measuring resting-state neural signal complexity with methods other than multiscale entropy revealed larger age-related changes at frontal and central regions compared to occipital ones (EEG measure:^106^; fMRI measure:^116^). However, we did not find an age-related decrease in resting-state alpha power (mainly at posterior electrode sites) which is quite unusual even in the eyes-open condition and could imply intact arousal and attention in these highly functioning older adults^139^.

The main question of our investigation was how adaptive training, undertaken to enhance cognitive control processes related to task-switching, could modify resting-state neural activity and complexity. Interestingly, these two EEG signal processing measures detected training-related changes in different experimental groups. While the spectral power density (SPD) showed similar training-related changes in both the young and old training-groups, multiscale entropy (MSE) changes were unique to the old-training group. Because the two methods measure linear and non-linear qualities of the neural function, they capture different processes, thus MSE could find more complex training-related effects which could not be detected by SPD^140,141^.

Our training-related resting-state SPD results revealed increased low frequency power in high delta (3–4 Hz), theta (4–8 Hz) and alpha (8–14 Hz) bands after training mostly around central channels in both the young and old training-groups (more midline and right centro-parietal changes for delta and theta bands and more fronto-central changes for alpha band). The MSE results showed increases in coarse scale (20–50 ms) brain signal complexity by training mostly at midline and right fronto-central electrode sites, exclusively in the old-training group. These results suggest that in younger adults, only the magnitude of the large-scale resting-state networks’ oscillatory activity (SPD) is altered by the task-switching training possibly because their brain dynamics and neural processing capacities are optimal. However, in older adults, adaptive training resulted in more complex changes in brain dynamics (MSE), possibly because older individuals were lower-performing at baseline on the task-switching paradigm (in reaction time, error rate and mixing costs, see results in^27^), therefore benefited more from training as they had more room for improvement than high-performing healthy younger adults (i.e., compensation account of cognitive training^142^).

In the old-training group, adaptive training increased coarse scale MSE and low frequency power from pre- to post-training, and modulated the age-related shift from more global and large-scale to local neural processing in resting-state brain dynamics for training-related regions. This increase in global network dynamics was most pronounced at midline and right fronto-central channels. The importance of fronto-central areas in age-related neural dynamics difference was established by Sleimen-Malkoun and colleagues^91^, who detected the largest age-related difference in coarse scale entropy at these fronto-central electrode sites (more complex neural signals in young adults). Moreover, other entropy-based measures of EEG signal complexity^106^ suggest that the most pronounced signal complexity decrease occurs at frontal and central electrode sites with aging, but this complexity decrease is slower in the right hemisphere. In line with these results, the more intact dynamics in the right fronto-parietal regions appear to support compensatory mechanisms in cognitive aging which are connected to different cognitive processes like alertness, sustained attention, response to novelty, self-monitoring and working memory^143^. Therefore, our results are consistent with the idea that for older individuals, the influence of cognitive training on coarse scale neural dynamics and complexity is most crucial at right fronto-centro-parietal areas.

In the current study, we did not indentify training-related changes in power at high frequencies and only a small increase in the fine scale MSE in the old-training group. Increased local information processing seems to be necessary to maintain^93^ and achieve^53,92^ better cognitive performance in older people (however, in order to achieve better cognitive performance, simultaneously increased long-range processing could be important as well^53,92^). The less distributed but still significant increase from pre- to post-training in fine timescale MSE (1–20 ms) in the old-training group was detected at midline and left fronto-central channels (mostly at F3, Fz and Cz). Despite the low spatial resolution of EEG measures, this area, mainly the left inferior frontal junction, was found to be crucial in task-switching performance and cognitive flexibility through task rule representation and task-switch mediation measured with task-related fMRI^144,145^. Moreover, increased neural signal variability in this area (measured with BOLD-signal variability) facilitated cognitive flexibility, better task-switching performance, and resistance to irrelevant distraction^146,147^. Therefore, training-evoked increases in local resting-state brain signal complexity at left fronto-central channels would support increased local network dynamics in task-specific regions in the old-training group, which remained present even during rest.

Additionally, we found a training-related power increase mostly in theta (largest increase at midline and right centro-parietal areas) and alpha (largest increase at fronto-central areas) frequency bands in both the young- and old-training groups. Earlier studies connected theta oscillatory activity to cognitive control processes^87^ and they found positive association between theta power mostly at midfrontal and parietal brain areas. Theta oscillatory activity has also been linked to performance on executive function tasks^100,148,149^ and multitasking training connected to cognitive control processes^150^. Alpha oscillatory activity has been linked to increased power during internally directed attention and during the inhibition and timing of specific cortical regions’ activation mainly in frontal and parietal areas^87,151^. Moreover, both theta and alpha oscillations are connected to cognitive performance^152^ and a combined neurofeedback and training study showed increased frontal theta and alpha activity with improved task performance^153^. During resting-state, Clements and colleagues^154^ showed that resting alpha power is connected to proactive control processes (maintenance of currently active representations), while resting theta power is related to reactive control (updating of representations and resolving interferences) in both younger and older adults. Based on these previous findings, the training-related resting-state increase mainly in theta and alpha power may be related to the increased internally oriented attention to the trained task and enhanced cognitive control processes and performance in both of our young- and old-training groups. These training-related changes were detected by improved attention orientation measured with Attention Network Test, and enhanced target-locked P3b component (which is connected to working memory updating^155^ and decision monitoring^156^) in both training groups, while better task performance and increased target-locked N2 amplitude (which reflects cognitive control^157^ and attention switching^158^) was found in the old-training group in the original training study^27^.

Despite the novelty of the presented work, certain limitations exist. One limitation is that we used passive control instead of an active one in the original experimental design. Thus, we are unable to separate pure adaptive training effects from the effect of active and repeated engagement of a control task and increased familiarity with the experimental environment in our brain dynamics measures and information processing changes. However, because our results are robust and in line with previous literature, they still present important information about training and age-related changes in general and intrinsic brain dynamics more specifically. The generalizability of our findings is not ideal, because only women participated, and the older group was highly educated: older participants had significantly higher IQ scores than younger participants (see^27^). This pattern is a general problem in healthy aging studies since older adults who are willing to participate in EEG studies are often highly educated, and physically and mentally active. Thus, it would be important to engage the general older population in these studies since it is debatable if more impaired cognitive processes in older people would lead to an even larger training-effect. That is, greater improvement capacity (compensation account of cognitive training) may support greater training-related improvement, but lower baseline cognitive abilities may undermine potential training-related change (related to the magnification account of cognitive training)^159^.

In conclusion, we identified general, adaptive training-related changes in information processing capacity and neural dynamics. While in the younger age-group adaptive task-switching training generated only linear and less complex changes (identified with spectral power density), in the older age-group both linear and more complex non-linear neural dynamics (identified with multiscale entropy) were affected. Namely, the general age-related information processing shift from more global to more local network communication was modulated by adaptive training: for older participants, adaptive training resulted in increased coarse timescale MSE and increased oscillatory activity in theta and alpha frequency bands. Thus, adaptive training may have a general and broad modulatory effect on cognitive and neural aging.

## Data Availability

The datasets for this study can be found at https://web.gin.g-node.org/gaalzs/TS_training_MSE_SPD.
